# The identification of hub genes associated with pure ground glass nodules using weighted gene co-expression network analysis

**DOI:** 10.1186/s12890-024-03072-z

**Published:** 2024-06-10

**Authors:** Yuan Cheng, Zuoqing Song

**Affiliations:** 1 Department of Lung Cancer Surgery, Tianjin Medical University General Hospital, Tianjin, 300052 China; 2 Department of Thoracic Surgery, North China University of Science and Technology Affiliated Hospital, Tangshan, Hebei 063000 China

**Keywords:** Pure ground glass nodules(pGGNs), Hub genes, WGCNA, Ribosome

## Abstract

**Background:**

Whether there are invasive components in pure ground glass nodules(pGGNs) in the lungs is still a huge challenge to forecast. The objective of our study is to investigate and identify the potential biomarker genes for pure ground glass nodule(pGGN) based on the method of bioinformatics analysis.

**Methods:**

To investigate differentially expressed genes (DEGs), firstly the data obtained from the gene expression omnibus (GEO) database was used.Next Weighted gene co-expression network analysis (WGCNA) investigate the co-expression network of DEGs. The black key module was chosen as the key one in correlation with pGGN. Gene Ontology (GO) and Kyoto Encyclopedia of Genes and Genomes (KEGG) pathways analyses were done. Then STRING was uesd to create a protein-protein interaction (PPI) network, and the chosen module genes were analyzed by Cytoscape software.In addition the polymerase chain reaction (PCR) was used to evaluate the value of these hub genes in pGGN patients’ tumor tissues compared to controls.

**Results:**

A total of 4475 DEGs were screened out from GSE193725, then 225 DEGs were identified in black key module, which were found to be enriched for various functions and pathways, such as extracellular exosome, vesicle, ribosome and so on. Among these DEGs, 6 overlapped hub genes with high degrees of stress method were selected. These hub genes include RPL4, RPL8, RPLP0, RPS16, RPS2 and CCT3.At last relative expression levels of CCT3 and RPL8 mRNA were both regulated in pGGN patients’ tumor tissues compared to controls.

**Conclusions:**

To summarize, the determined DEGs, pathways, modules, and overlapped hub genes can throw light on the potential molecular mechanisms of pGGN.

## Introduction

With the widely application of computed tomography with low dose, a gradually increasing number of pGGNs have been detected [1]. Known as ground glass nodules (GGNs), these nodules mainly exists in bronchial and vascular margins in the lung [2]. GGN can be further divided into part-solid GGN and pGGN, according to whether solid components are existed in the lesion [3]. If an invasive pGGN persist for a long term, an early malignant tumor may be associated with this condition, so it is crucial to distinguish the invasiveness of pGGNs [4]. Although a large number of pGGNs may be benign lesions, however, there are still some invasive malignancies, which are detected through surgical resection, and the proportion of which vary from 1.7 to 24.3% [5]. The best treatment time to intervene pGGN is to remove high-risk pGGN before it spreads to metastatic sites, however, it is limited due to clinical examination’s sensitivity [6]. AND, up to now, there are few studies that have used radiomic feature analysis, which is to predict histologic subtypes in a cohort that there are only pGGNs composed [5]. 

WGCNA is an efficient and convenient technique to process transcriptome data, because it can not only cluster the tightly connected genes into various modules, but also explore module correlations, and Identify traits that interest you [7]. The Search Tool for Retrieval interacting Genes (STRING) [8] online databases were employed to build a network of protein–protein interactions (PPIs).

This is a novelty research about pGGNs based on the method of bioinformatics analysis.A profile of GSE gene expression was reanalyzed in this study using 23 patients with pGGN and 10 controls. Then we used the ‘limma’ on SangerBox network to obtain DEGs, and WGCNA conducted this study to create a network of gene co-expression, black key module was chose. This module analyzed gene ontologies and KEGG pathways for enrichment.PPI networks were constructed in the black module using the STRING database. At last by cytoscape software using the method of different calculations, the top 10 hub genes in the black module were identified, and six overlapped hub genes were screened out.The objective of this study is to investigate and identify the potential biomarker genes for pGGN, in order to understand the role of associated genes in the development and progression of pGGN and identify relevant molecular markers with value for early diagnosis and targeted therapy.

## Methods and materials

### Sources of data

**Fig. 1:**
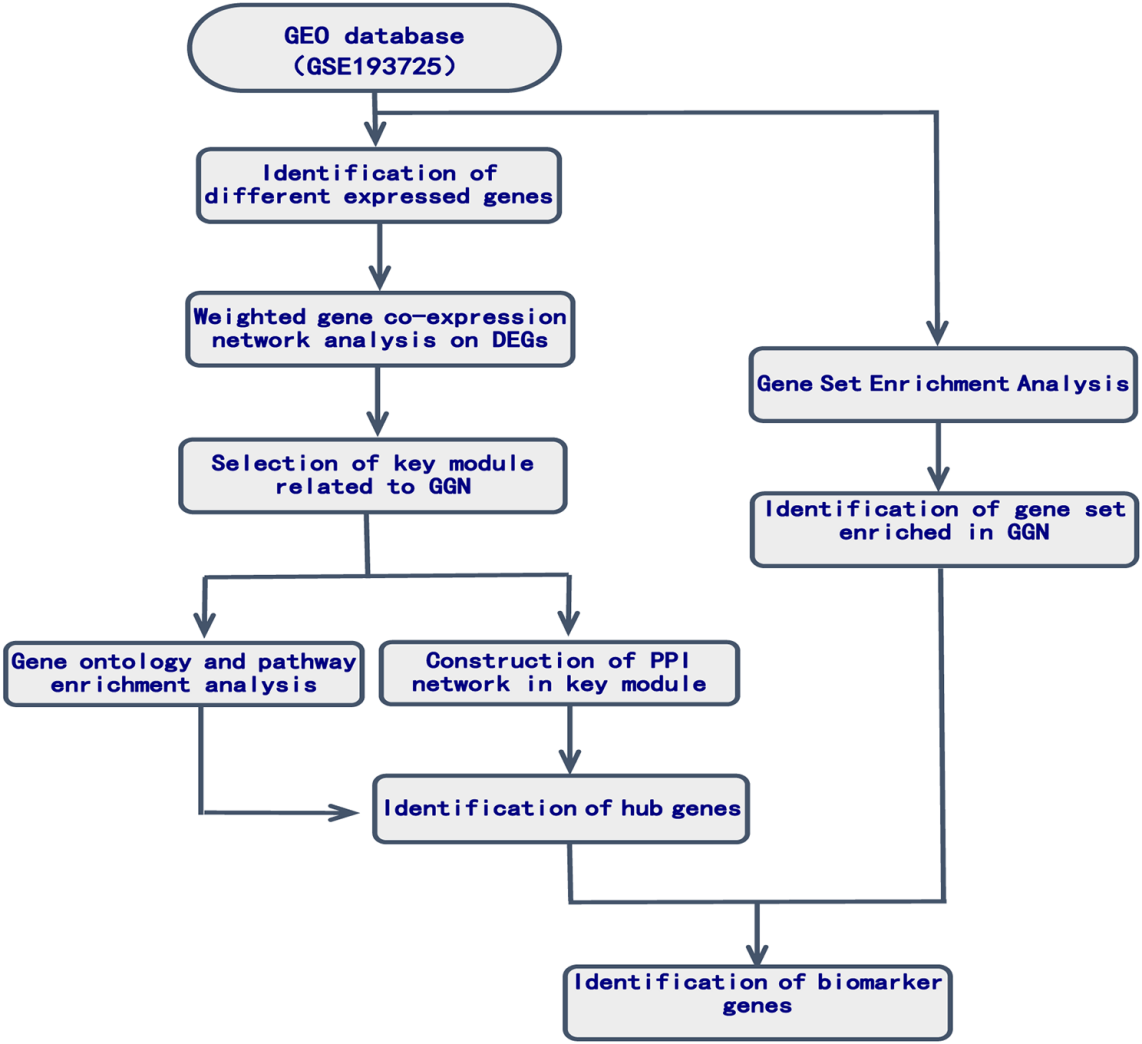
Workflow chart of the study

In Fig. 1 the workflow of the study was shown.An expression profile microarray GSE of mRNA transcripts, deposited by Nasser K. Altorki et al. [9], was retrieved from a free public database named Gene Expression Omnibus (GEO).It includes lung tissue from pGGN patients and controls.And on platform GPL24676 (GSE193725)the data were got, including 54 samples, 23 from pGGN patients, 10 from controls and 21 from solid nodule patients.In this study, the date of 23 pGGN patients and 10 controls were processed.

### DEGs identification

Firstly, data quality checks and log transformations were performed in order to cancel batches.Based on generalized linear models, Limma [10] is a method for screening differential expression data from microarrays, here we use limma (version 3.40.6) on SangerBOX to identify DEGs between the control and pGGN groups. For statistical significance, an adjusted p-value of 0.05 was used. For visualization of the top 50 DEGs, the Heatmap package was used to plot the volcano map.

### Key module identified by WGCNA

Based on the scale-free topology criteria, WGCNA [11] was employed to build the co-expression networks in DEGs. Firstly, all DEGs were analyzed in R software, and the soft thresholding power was determined. Next, the weighted co-expression network was constructed, and DEGs were clustered into several modules with different color labels. The correlation between each module and pGGN or controls was then explored. The module most correlated with pGGN was regarded as a key module for further enrichment analysis.

### Analysis of gene ontologies and pathways

With Metascape [12] transcriptome and genome data are efficiently invested in biological processes.In the black module both of GO [13] and KEGG [14] pathway analysis were dealt with. In order to visualize the terms for the functions and pathways, they were retrieved.

### PPI network and potential hub genes identification

The STRING [8] online database was used to create the PPI network in the black module.And the black key module employed an online database to build the PPI network, which was processed by Cytoscape software [15]. Cytoscape is an open source software project for integrating biomolecular interaction networks with high-throughput expression data and other molecular states into a unified conceptual framework.CytoHubba provide a user-friendly interface to explore important nodes in biological networks.Then Cytohubba [16] was used to get the hub genes, and alculate and draw custom vegn diagrams was used to get six overlapped hub genes.

### Gene set enrichment analysis(GSEA)

In order to identify whether the KEGG pathway is statistically significantly enriched in pGGN and control subjects, the GSEA [17] was conducted, whose desk application was used to import transcriptome data according to instructions on the website. The significant gene sets were determined by both *p* < 0.05 and FDR < 0.25.

### Hub gene-mediated signaling pathways

‘GeneMania’ [18] is used to predicate and identify the function of chosen hub genes. For the establishment of a putative PPI, the 10 top hub genes were imported into a database called ‘Genemania’. The results were processed and visualized.

### Case collection

To validate these hub genes, primary pGGN tissues and their corresponding adjacent noncancerous tissues were collected from Tianjin Medical University General Hospital, and patients were included if the following criteria were met: (1) preoperative chest HRCT examinations performed within 3 weeks; (2) tumor and control tissue specimens were complete and reviewable;3) P-pGGNs located next to the pleura on the preoperative HRCT, defined as pGGNs. Normal corresponding adjacent lung tumor tissues whose distance from the edge of tumor was more than 3 cm were regard as the controls. All samples were immediately frozen and saved in liquid nitrogen. Enrolled patients had not received neoadjuvant chemotherapy or radiotherapy. Patients underwent emergency surgery were excluded.

### Quantitative real-time polymerase chain reaction (qRT-PCR)

The total RNA in tissues was isolated using RNAzol® RT according to the instructions (GeneCopoeia, Rockville, MD, USA). And RNA was reverse transcribed into complementary DNA (cDNA) using reverse transcription kit (Thermo, USA). The real-time quantitative PCR was performed according to the instructions of SYBR GREEN kit (TaKaRa, Japan) with Roche LightCycler480® Probes Master reagent (Roche, Basel, Switzerland). GAPDH served as internal reference control. The relative expression levels of genes were calculated by the 2^−ΔΔCt^ method. All primers used were synthesized by Sangon Biotech (Shanghai, China) and the sequences were listed in Table 1.

**Table 1:** Primer sequences for quantitative real-time PCR

Gene	Primer Sequence (5’→3’)	
GAPDH	Forward	GGAAGCTTGTCATCAATGGAAATC
	Reverse	TGATGACCCTTTTGGCTCCC
CCT3	Forward	TCAGTCGGTGGTCATCTTTGG
	Reverse	CCTCCAGGTATCTTTTCCACTCT
RPL4	Forward	GCCAGGGTGCTTTTGGAAAC
	Reverse	GGGCAGAACAGATGGCGTAT
RPL8	Forward	AGAAGACCCGTGTGAAGCTG
	Reverse	GGTTTGTCAATTCGGCCACC
RPLP0	Forward	TGGGCAAGAACACCATGATG
	Reverse	CGGATATGAGGCAGCAGTTTC
RPS16	Forward	GTGTCCGTGTAAAGGGTGG
	Reverse	ACTGGATGAGGATGTCTTTGATC
RPS2	Forward	GCCAAGCTCTCCATCGTC
	Reverse	GTGCAGGGATGAGGCGTA

### Statistical analysis

All experiments were performed in triplicate, and data were pooled from three independent experiments. Data of gene expression analysis was expressed as mean ± standard deviation (SD) and estimated using Mann–Whitney nonparametric test. All the rest of the experiments were used paired t-test or one-way ANOVA test. T-test was used for the comparison of composition ratio between groups. SPSS 22.0 software (SPSS, Chicago, IL, USA) and Graphpad Prism version 8.0 (GraphPad Software Inc, La Jolla, Calif) was used for statistical analysis. *P*<0.05 was considered statistically significant.

## Results

### Identifications of DEGs

**Fig. 2:**
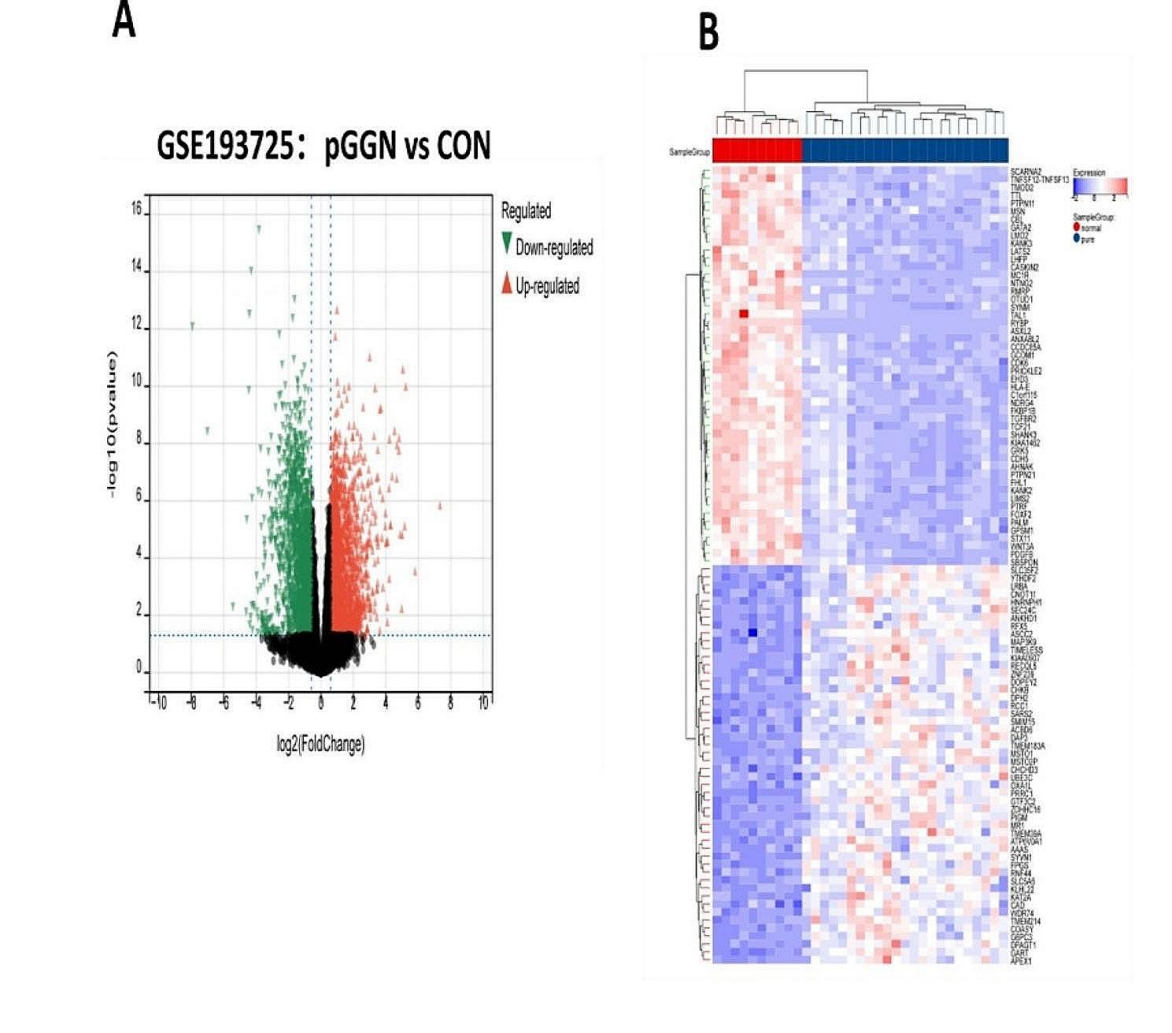
Expression profile of DEGs. **(A)** Volcano map of DEGs expression levels. **(B)** Heatmap of top 50 DEGs.

There were totally 4475 DEGs identified between pGGN patients and controls, with an adjusted p-value of < 0.05. In pGGN, 2415 DEGs were upregulated and 2060 were downregulated. Figure 2A shows the volcano map of almost all DEGs, and Fig. 2B displays the heatmap of the top 50 DEGs.

### WGCNA analysis

**Fig. 3:**
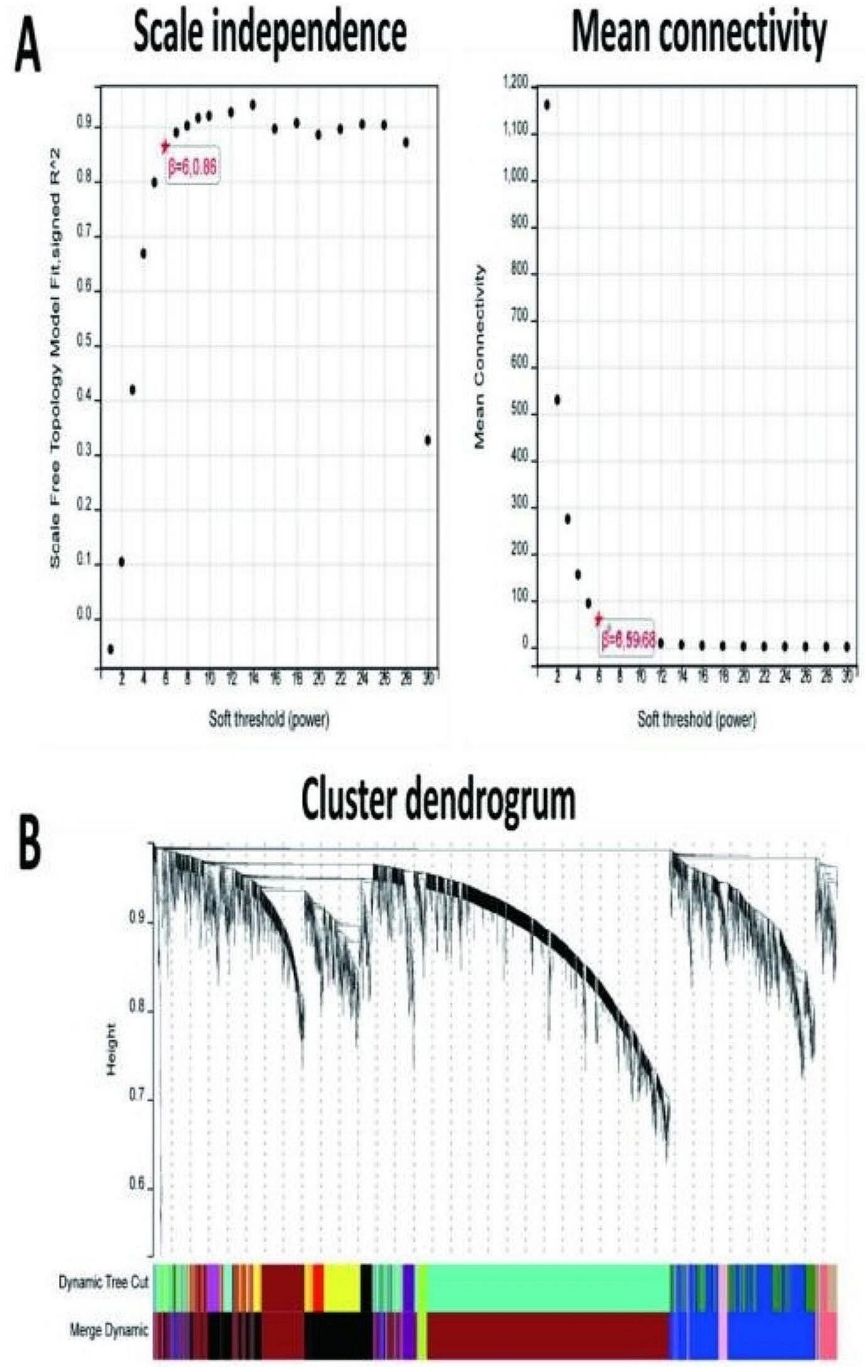
WGCNA of DEGs. **(A)** Estimation of the soft thresholding value for a scale-free co-expression network. **(B)** Cluster dendrogram of all DEGs.

**Fig. 4:**
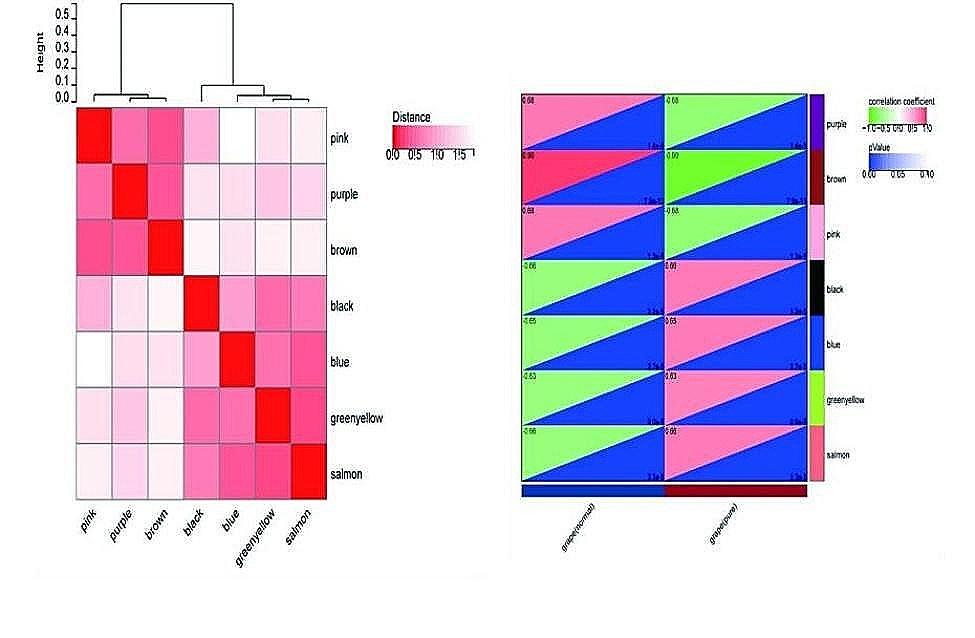
**(A)** Module feature vector. **(B)** Correlation between each module and pGGN patients or controls

Further processing was performed on the 4475 DEGs identified with the WGCNA package in R software on SangerBOX, and a scale-free co-expression network was established.As part of the subsequent analysis, a soft thresholding power of 6 was chosen.The soft thresholding power β was set at 6 in the subsequent analysis, because the scale independence reached 0.8(Fig. 3A) and had a relatively good average connectivity.It was found that these DEGs were totally clustered into seven different modules(Fig. 4A), black, brown, blue, greenyellow, pink, purple, andsalmon, Using a minimal module size of 30. Figure 3B showed the cluster dendrogram of these DEGs.And Fig. 4B showed the correlation between pGGN and each module. The results indicated that black(0.66, *p* < 0.0001) model was one of the most positive module and brown (-0.90, *p* < 0.0001) was the most negative module. Then the most upregulated black module was chosen, which included a total of 590 DEGs, as the key module related to pGGN.

**Fig. 5:**
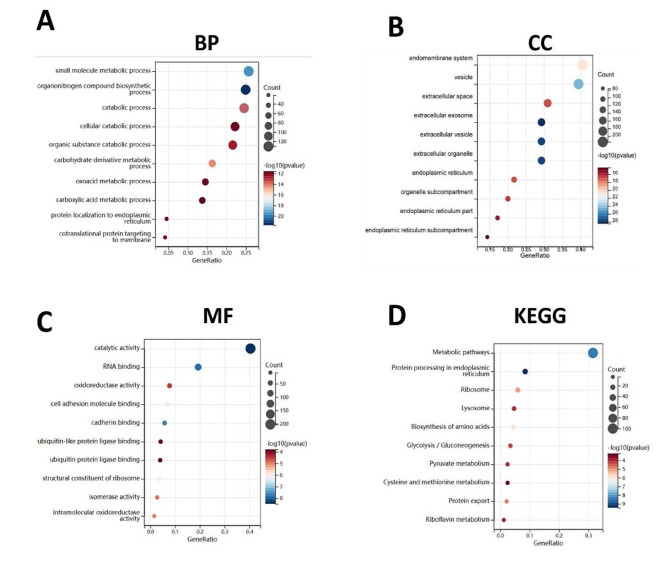
Gene ontology and KEGG enrichment analysis. **(A)** Biological process. **(B)** Cellular component. **(C)** Molecular function. **(D)** KEGG enrichment analysis

**Fig. 6:**
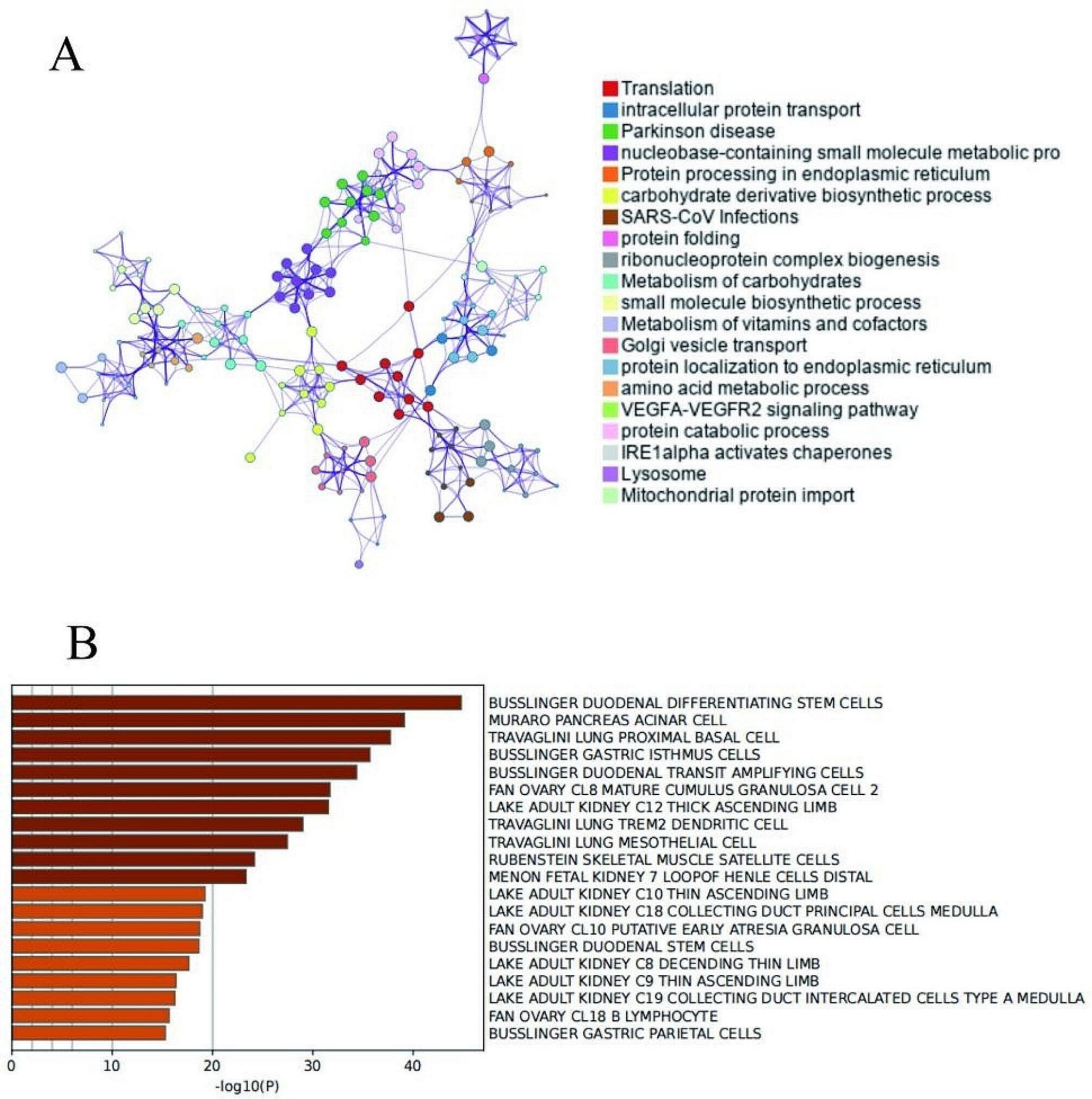
**(A)** Network of enriched terms **(B)** Bar graph of enriched terms

Using Metascape, 590 DEGs in the black module were analyzed for GO and pathway enrichment.The result showed that all DEGs in the black module were found enriched in 52 molecular functions (MF), 169 cellular components (CC), and 422 biological processes (BP). Figure 5A–C showed the top 10 BPs, CCs, and MFs. Figure 6A-B showed the result of Metascape tool pathway enrichment analysis. The GO category showed that ‘endomembrane system,’ ‘vesicle,’ and ‘extracellular space’ were enriched markedly.And KEGG analysis revealed that the black module was enriched in 24 pathways, such as ‘Lysosome,’ ‘Ribosome,’ ‘Protein processing in endoplasmic reticulum’ (Fig. 5D), and so on.

### GSEA analysis

**Fig. 7:**
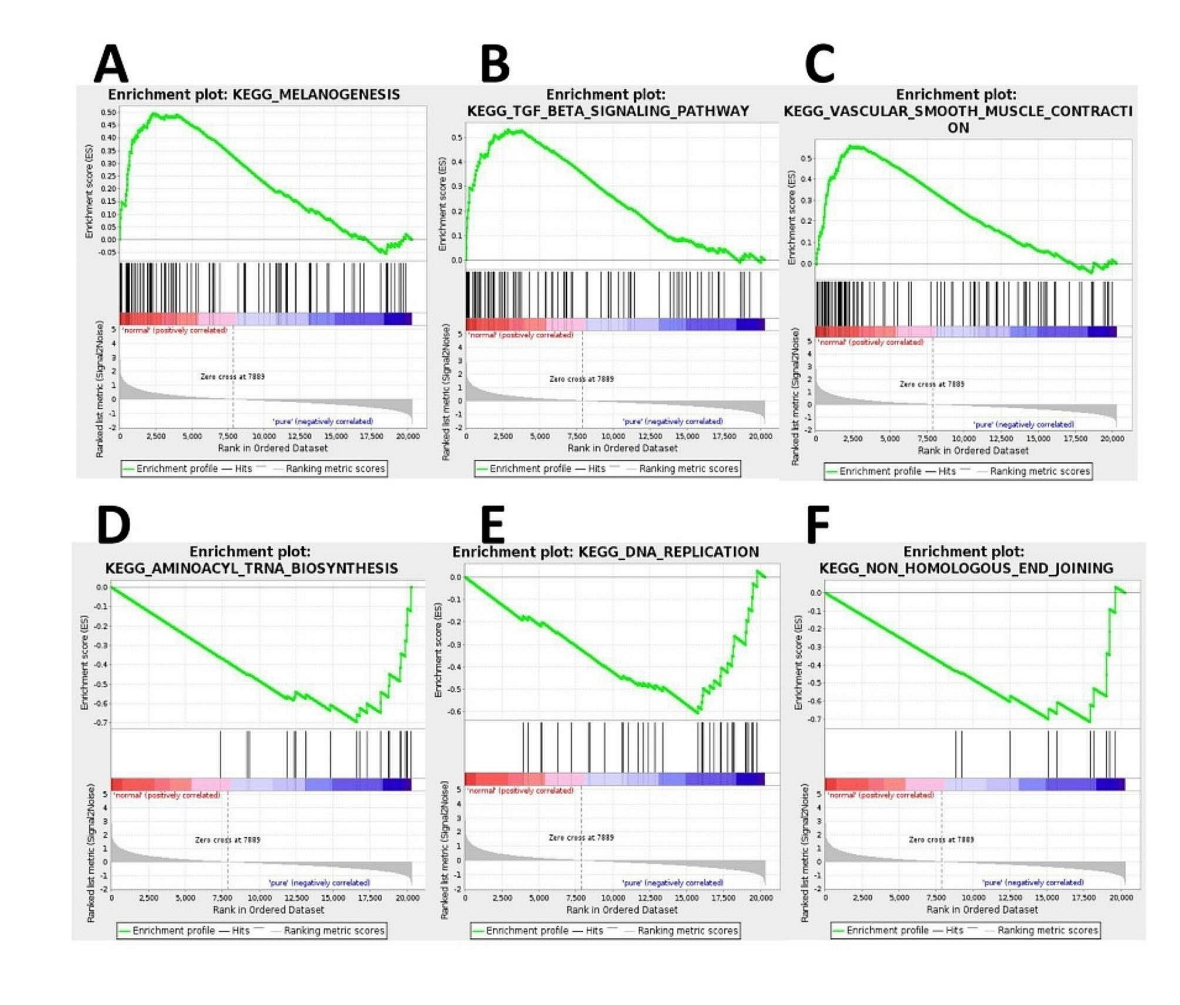
Gene set enrichment analysis. **(A)** Enrichment plot of ‘MELANOGENESIS’with enrichment score 0.50, FDR q-value 0.0. **(B)** Enrichment plot of ‘TGF_BETA_SIGNALING_PATHWAY’with enrichment score 0.53, FDR q-value 0.0. **(C)** Enrichment plot of ‘VASCULAR_SMOOTH_MUSCLE_CONTRACTION’ with enrichment score 0.56, FDR q-value 0.002. **(D)** Enrichment plot of ‘AMINOACYL_TRNA_BIOSYNTHESIS’ with enrichment score − 0.70, FDR q-value 0.012. **(E)** Enrichment plot of ‘DNA_REPLICATION’ with enrichment score − 0.61, FDR q-value 0.001. **(F)** Enrichment plot of ‘NON_HOMOLOGOUS_END_JOINING’ with enrichment score − 0.72, FDR q-value 0.001

With GSEA software, it was explored how pathway gene sets were distributed among the pGGN patients and controls.The results demonstrated that ‘vascular_smooth_muscle_contraction’ was the most remarkably enriched in the pGGN group, and Fig. 5A showed its enrichment score is 0.55, suggesting that ‘vascular_smooth_muscle_contraction’ may play an important role in the pathophysiology of pGGN patients.And Fig. 7A–F showed the top up-regulated and down-regulated gene sets.

### PPI network construction and overlapped hub gene analysis

**Fig. 8:**
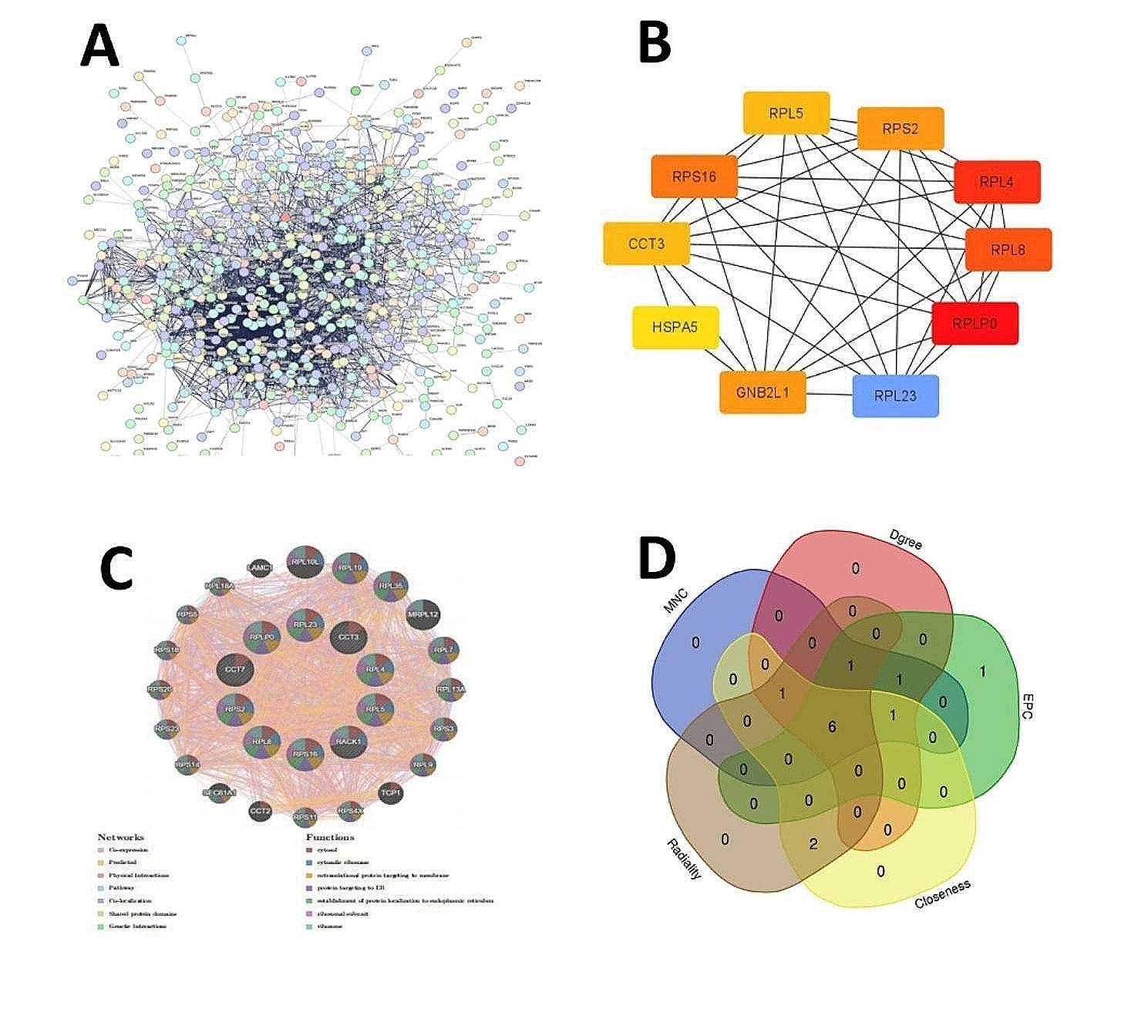
PPI network and hub gene. **(A)** PPI network. **(B)** The top 10 genes ranked by Dgree. **(C)** The top 10 degree genes analyzed in GeneMANIA. **(D)** The overlapped hub genes from different algorith

STRING database was used to explore the interaction between these genes in the black module. And the STRING PPI network was constructed by setting 0.4 as the minimum interaction score. Figure 8A showed that the PPI network included totally 587 nodes and 3810 edges. The top ten hub genes (Table 2) were screened out by twelve different calculation algorithms, such as MNC, MCC, EPC, DMNC, Degree, and so on, Using the cytoHubba plug-in in Cytoscape. The top 10 genes ranked by Degree calculation algorithms were analyzed in GeneMANIA (Fig. 8B-C).And six overlapped hub genes(Fig. 8D), including CCT3, RPL4, RPL8, RPLP0, RPS16, RPS2 were identified, using calculate and draw custom vegn diagrams, which were among the five algorithms (MNC, Degree, EPC, Closeness, Radiality).

**Table 2:** Top ten hub genes obtained by eleven algorithms of Cytohubba

DMNC	MNC	Degree	EPC	BottleNeck	EcCentricity	Closeness	Radiality	Betweenness	Stress	ClusteringCoefficient
EIF3M	RPLP0	RPLP0	RPL4	CDH1	XBP1	RPLP0	HSPA5	CDH1	HSPA5	HSPA5
RPS27L	RPL4	RPL4	RPLP0	XBP1	HSPA5	GNB2L1	GNB2L1	HSPA5	XBP1	XBP1
RPLP1	RPS16	RPL8	RPS16	GNB2L1	HSP90B1	HSPA5	HSPD1	GNB2L1	GNB2L1	GNB2L1
EIF6	RPL8	RPS16	RPL8	KDM1A	SSR3	RPL8	RPLP0	HSPD1	CDH1	CDH1
EIF3H	RPS2	GNB2L1	RPS2	CLTC	SRPRB	HSPD1	RPL8	XBP1	HSPD1	HSPD1
PSMD11	GNB2L1	RPS2	CCT5	CCT3	KRTCAP2	RPL4	RPL4	CLTC	SEC61A1	SEC61A1
EIF3I	RPL5	CCT3	RPL5	RPL23	PKM	CCT3	CCT3	SEC61A1	RPLP0	RPLP0
RPL17	RPL23	RPL5	CCT7	SNRPG	GPX4	RPS16	RPL23	CCT3	CCT3	CCT3
IFRD2	CCT7	RPL23	CCT3	HSP90B1	MRPL24	RPS2	CCT7	TUFM	TUFM	TUFM
RPLP2	CCT3	CCT7	GNB2L1	RPL8	WDR12	RPL23	RPS2	ALDH18A1	RPL8	RPL8

### Validation of the hub genes

**Fig. 9:**
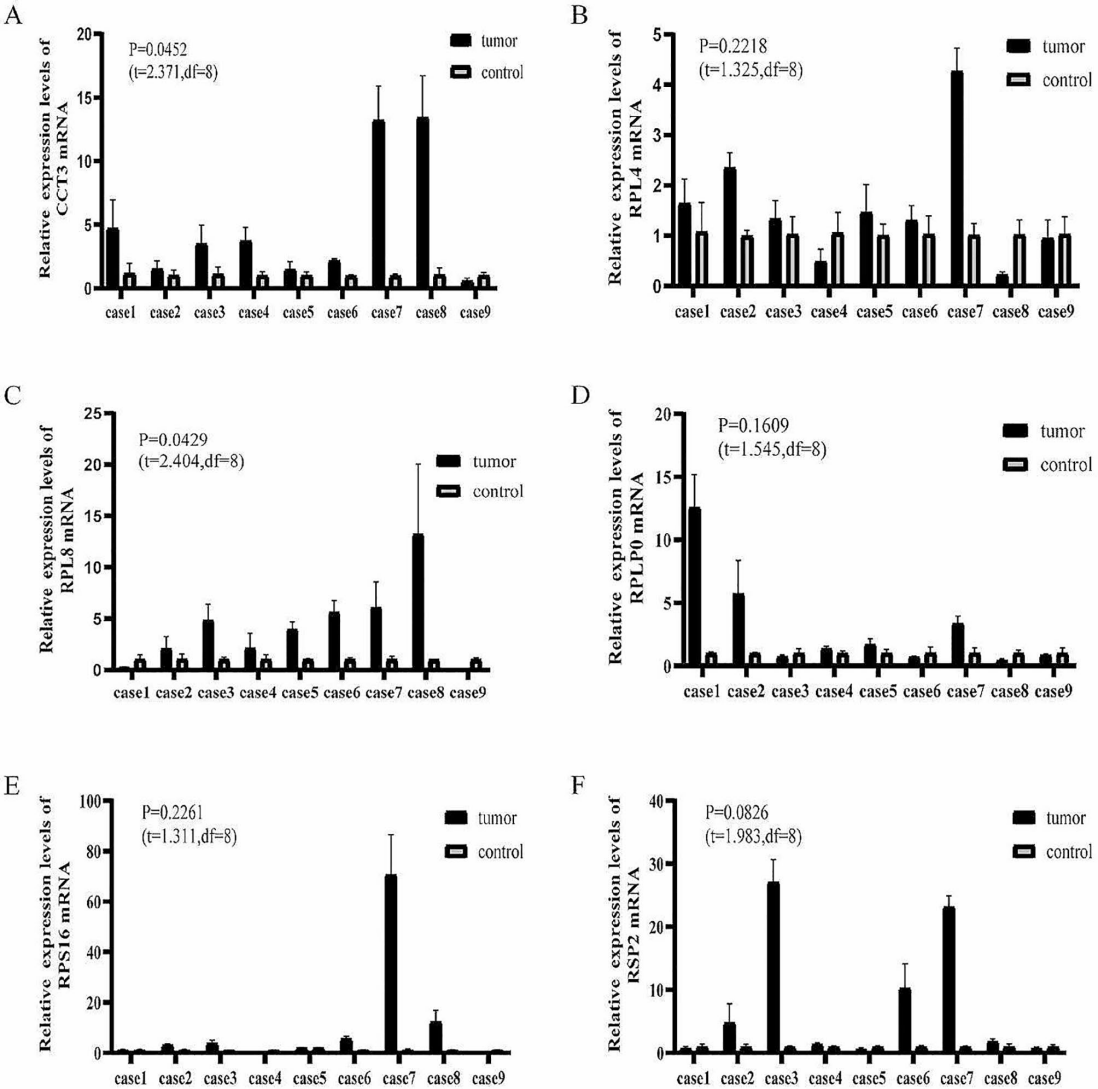
validation of six hub genes.The mean of differences(control-tumor) and SD of differences were shown as follow. **(A)** Relative mRNA levels of CCT3 in control tissues vs. pGGN tissues(mean, SD:-3.895, 4.928). **(B)** Relative mRNA levels of RPL4 in control tissues vs. pGGN tissues(mean, SD:-0.5307, 1.202). **(C)** Relative mRNA levels of RPL8 in control tissues vs. pGGN tissues(mean, SD:-3.228, 4.028). **(D)** Relative mRNA levels of RPLP0 in control tissues vs. pGGN tissues(mean, SD:-2.004, 3.968). **(E)** Relative mRNA levels of RPS16 in control tissues vs. pGGN tissues(mean, SD:-9.939, 22.74). **(F)** Relative mRNA levels of RSP2 in control tissues vs. pGGN tissues(mean, SD:-6.844, 10.35)

The transcriptional changes of six overlapped hub genes were detected in the lung tumor tissues and controls from the 9 pGGN patients by quantitative RT-PCR. The results indicated that CCT3 and RPL8 were more highly expressed in pGGN tissues than those in adjacent tissues(*P*<0.05) (Fig. 9A-F), showing that the identified hub genes CCT3 and RPL8 demonstrated a powerful discrimination capability as potential biomarkers for pGGN.

## Discussion

Nowadays adenocarcinoma, one subtype of lung cancers, is the most common histologic type, which always appears as localized GGOs on CT, and a pure ground glass nodule is likely a preinvasive lesion [19]. Although there are several types of pGGNs, all of which are slow-progressing diseases, but they can show differing growth patterns and degrees of invasion, such as adenocarcinoma in situ (AIS), minimally invasive adenocarcinoma (MIA), and even invasive adenocarcinoma (IAC) [20]. IF the pGGN is AIS or MIA, a small wide wedge dissection would be enough without lymph node, but a small peripheral IAC need segmentectomy, which may be more suitable [5]. So it is crucial to elucidate the effective diagnostic markers of pGGN.

There is little previous bioinformatics study of pGGNs.This study used the GEO database to download mRNA expression profile GSE193725 and then perform a WGCNA on it. Using the novel approach, all 4475 DEGs were obtained by the ‘limma’ on SangerBox network, all these DEGs were then clustered into seven modules. And the black module, which included 590 genes, was chosen to be the most upregulated module closely related to the pGGN(whose correlation score is 0.66,and *p* < 0.0001). All of these identified genes were enriched in ‘Lysosome,’ ‘Ribosome,’ and ‘metabolic pathways’ mainly. GSEA showed that ‘vascular_smooth_muscle_contraction’ was markedly in the pGGN group. Six overlapped hub genes, CCT3, RPL4, RPL8, RPLP0, RPS16, RPS2, were identified by cytoscape software. And it was found the identified hub genes CCT3 and RPL8 demonstrated a powerful discrimination capability as potential biomarkers for pGGN.

using mRNA as a template, and using amino acids as raw materials to synthesize proteins is the ribosome’s primary function [21]. Previous studies have showed that the ribosome has a great deal of functions, including affecting protein synthesis and taking a significant role in cell differentiation, proliferation, transformation and apoptosis [22]. The ribosome biogenesis dysregulation was important in cancer because it identifies nucleoli with irregular shapes and numbers [23]. And ribosome biogenesis hyperactivation addiction in cancer cells is clear, by illustrating its numerous molecules as cancer treatments through impairing ribosome production [24]. In normal cells, ribosomes are involved in proliferating by synthesizing proteins, but in cancer cells, ribosomes are needed according to metabolic requirements. And what’s more, previous study has reported that cancer cells express about nearly 10,000 different proteins [25]. Ribosome biogenesis can be a target in cancer cells because of three main reasons, firstly in certain cancer types and its cell populations, this process is highly active and essential, secondly several chemotherapy drugs have been already proven to exert their some pharmacological effects partly, through impairing ribosome biogenesis, and last but not the least blocking ribosome biogenesis can lead in a lot of cases to the activation of p53 [26]. 

RPL4 could be bound by PRDX2, being reduced the interaction between MDM2, resulting in promoting the proliferation of colorectal cancer cells [27]. RPL8 is reported that with pancreatic tumor grades it alters significantly, which may predict a differential expression prognosis of pancreatic cancer [28]. RPLP0 suppressed by the upregulated level of miR-4731-5p, can be a target in non-small-cell lung cancer [29]. And for advanced hepatocellular carcinoma cells, RPS16 may be a potential novel target [30]. PRS2 identified by hub genes was lowly expressed in rats with pulmonary arterial hypertension [31]. In combination with the previous study, we suppose that RPS2 and CCT3 may regulate the proliferation of cells in the pathological process of pGGN. Nevertheless, further research on the role of RPS2 and CCT3 is required to validate the relationship.

To sum up, Using weighted gene co-expression network analysis we found that RPL4, RPL8, RPLP0, RPS16, RPS2 and CCT3, have the powerful ability to screen out pGGN from the controls.And through the detection by quantitative RT-PCR, CCT3 and RPL8 demonstrated a powerful discrimination capability as potential biomarkers for pGGN. But it is still required that more sample size is needed to validate the efficacy of these overlapped genes as biomarkers for pGGN in the immediate future.

## Conclusions

To conclude, it was re-analyzed the expression profile GSE193725 with WGCNA. And it was screened out six overlapped hub genes in pGGN, including RPL4, RPL8, RPLP0, RPS16, RPS2 and CCT3. However, research on the role of these hub genes in pGGN remains limited. The results show that RPL4, RPL8, RPLP0, RPS16, RPS2 and CCT3 can be served as potential target biomarker genes for diagnosis for pGGN. And CCT3 and RPL8 demonstrated a powerful discrimination capability as potential biomarkers for pGGN.

## Data Availability

The mRNA expression dataset used in our study was downloaded from the Gene Expression Omnibus (GEO) under accession number GSE193725 (https://www.ncbi.nlm.nih.gov/geo/query/acc.cgi? acc=GSE, accessed on 22 March 2023).
